# Hidden reservoir of resistant parasites: the missing link in the elimination of falciparum malaria

**DOI:** 10.1186/s40249-016-0227-5

**Published:** 2017-02-06

**Authors:** Rashad Abdul-Ghani, Mohammed A. K. Mahdy, John C. Beier, Leonardo K. Basco

**Affiliations:** 1 Department of Parasitology, Faculty of Medicine and Health Sciences, Sana’a University, Sana’a, Yemen; 2 Tropical Disease Research Center, University of Science and Technology, Sana’a, Yemen; 3 Department of Public Health Sciences, University of Miami Miller School of Medicine, Miami, FL USA; 4 Unité de Recherche 198, Unité de Recherche sur les Maladies Infectieuses et Tropicales Emergentes, Institut de Recherche pour le Développement, Faculté de Médecine La Timone, Aix-Marseille Université, Marseille, France

**Keywords:** Malaria, *Plasmodium falciparum*, Submicroscopic infection, Drug resistance, Elimination

## Abstract

**Background:**

To successfully eliminate malaria, an integrated system that includes a number of approaches and interventions—aimed at overcoming the threat of antimalarial drug resistance—is required. Significant progress has been made in reducing malaria incidence through large-scale use of artemisinin-based combination therapies and insecticide-treated nets. To consolidate these gains, attention should be paid to the missing links in the elimination of malaria. One of these gaps is the residual reservoir of submicroscopic resistant parasites, which remains after case management or other control measures have been carried out. Therefore, the present opinion piece highlights the importance of exploring the role that submicroscopic resistant parasites could play in hindering malaria elimination by allowing the persistence of transmission, particularly in areas of low transmission or in the pre-elimination and/or elimination phase.

**Discussion:**

If malaria elimination interventions are to be effective, the relative role of the hidden reservoir of resistant parasites needs to be assessed, particularly in regions that are low-transmission settings and/or in pre-elimination and/or elimination phases. Various ongoing studies are focusing on the role of submicroscopic malaria infections in malaria transmission but overlook the possible build-up of resistance to antimalarial drugs among submicroscopic parasite populations. This is an important factor as it may eventually limit the effectiveness of malaria elimination strategies.

**Conclusions:**

An evidence-based estimation of the “true” reservoir of resistant parasites can help target the existing and emerging foci of resistant parasites before they spread. Emergence and spread of artemisinin-resistant *Plasmodium falciparum* malaria in Southeast Asia underline the need to contain drug resistance.

**Electronic supplementary material:**

The online version of this article (doi:10.1186/s40249-016-0227-5) contains supplementary material, which is available to authorized users.

## Multilingual abstracts

Please see Additional file 1 for translations of the abstract into the five official working languages of the United Nations.

## Background

Globally, countries and regions affected by malaria are devoting efforts to gradually eliminate it for good. Since 1998, great strides have been made in the fight against malaria with the launch of the globally coordinated initiative, the Roll Back Malaria Partnership, by the World Health Organization (WHO), the United Nations (UN) Children’s Fund, the UN Development Programme, and the World Bank [1]. The UN’s latest Millennium Developmental Goals Report raises hopes as it documents a drop in the overall rates of global malaria incidence and mortality by about 37 and 58%, respectively, as well as the prevention of over six million deaths, between 2000 and 2015 [2]. The global decrease in the malaria burden is mainly attributed to the introduction of artemisinin-based combination therapies (ACTs) and insecticide-treated nets (ITNs); more than 900 million ITNs have been distributed in sub-Saharan African countries in the last ten years [2]. However, this does not mean that the mission has been accomplished [3]. Increasing malaria resistance to non-artemisinin partner drugs is alarming and necessitates rapid action in order to maintain frontline ACTs [4]. The emergence and spread of artemisinin resistance as well as the failure of its containment across Southeast Asia may reverse the significant advances made in falciparum malaria control thus far [5–7]. It is noteworthy that ACT- and ITN-based interventions may result in the infectious reservoir in low-transmission settings to be predominantly submicroscopic [8]. Consequently, efforts should be dedicated to detecting submicroscopic drug-resistant parasite populations in regions that are low-transmission settings and/or in pre-elimination phases. Combating malaria should be oriented towards the realization of the Sustainable Development Goal of the UN to *“ensure healthy lives and promote well-being for all at all ages*” in endemic, resource-limited countries [9, 10], and such control and elimination efforts must consider the hidden reservoir of resistant parasites.

With the absence of a clinically effective vaccine against malaria [11–13], prompt diagnosis and timely treatment with efficacious antimalarial drugs is still one of the principal mainstays of malaria control and elimination. The WHO recommends that treatment should be given after diagnosis by microscopy or rapid diagnostic tests [14]. However, despite this process being important for the prevention of severe malaria, treatment of laboratory-confirmed malaria overlooks the asymptomatic and submicroscopic malaria infections that could potentially contribute to parasite transmission and the spread of drug resistance. The use of mass drug administration (MDA) is still controversial as an effective strategy for malaria elimination due to concerns about the possibility of accelerating drug resistance and faces a number of operational challenges, including coverage, safety and monitoring its impact on the rate of transmission [15]. In addition, it is difficult to persuade everyone to take medications in MDA campaigns when they are not sick. However, even if this approach was applied to a certain geographical area, there still would be a need to assess the resistance situation among residual submicroscopic parasites, taking into account that partially effective treatment may leave resistant parasites behind. Therefore, the present opinion paper discusses this missing link of submicroscopic resistant parasites that form a hidden reservoir of resistance to antimalarial drugs that needs to be assessed for possible role in persistent transmission. In addition, it highlights the importance of assessing its magnitude and potential in hindering malaria elimination, particularly in areas of low transmission or in pre-elimination phase, to take the necessary steps in order to interrupt the spread of malaria and antimalarial drug resistance.

## Discussion

The hidden reservoir of resistance to antimalarial drugs refers to the submicroscopic reservoir of resistant malaria parasites that usually circulate in low-endemicity settings, particularly the residual parasites remaining after treatment. One of the greatest challenges that hinders malaria elimination in areas of low-transmission or in those entering the pre-elimination phase is the residual *Plasmodium falciparum* parasites that are often submicroscopic, partly because of drug resistance, particularly in areas where artemisinin-resistant parasites have emerged. Intuitively, submicroscopic malaria infections are resistant so that they can persist in such situations. Most clinical and epidemiological studies on antimalarial drug resistance have focused on microscopically patent infections, which makes it difficult to determine the true burden of resistance in pre-elimination and elimination settings, as this approach ignores the hidden reservoir of submicroscopic resistant *P. falciparum* genotypes in surveillance studies for the detection of molecular markers of resistance. This is of paramount importance as residual resistant parasites could contribute to the risk of further malaria transmission, or even be the cause of a resurgence or spread of malaria.

## Reservoir of submicroscopic malaria infection and resistant parasites: the need to shift gears from sensitive to ultra-sensitive detection

The detection of drug resistance in submicroscopic malaria infections requires the development of ultra-sensitive molecular techniques. The nested polymerase chain reaction (PCR), which amplifies 18S rRNA, has a detection limit of six parasites/μl of blood from dried spots blotted onto filter papers [16]. Molecular assays that have been developed more recently have a detection limit of 0.02 parasite/μl (by quantitative PCR) and 0.01 parasite/μl (by quantitative nucleic acid sequence-based amplification assays) [17]. The field-applicable loop-mediated isothermal amplification (LAMP) has a detection limit of 0.1–2 parasites/μl [17, 18]. Although molecular techniques can be used in retrospective epidemiological surveys as they involve the collection of blood samples on filter paper, these methods use small volumes of finger-prick capillary blood (usually 5 μl), which limit their detection potential in very low parasite densities. Supposing that a technique could amplify nucleic acids from a single parasite, its sensitivity would still be limited due to the small sample volume (one parasite per 5 μl) and it could not detect parasite densities of <200 parasites/ml [19]. Generally, the detection limit of using finger-prick capillary blood is 1 000 or more parasites per ml [19]. To address this challenge, quantitative real-time PCR methods using increased venous blood volumes (0.5–1.0 ml) have been developed and validated to detect parasite densities as low as 20–22 parasites/μl [19, 20].

Interruption of malaria transmission through MDA or mass screen-and-treat strategies necessitates the development of next generation rapid diagnostic tests that can detect the lowest threshold of infectiousness of human populations to vector mosquitoes [21]. In epidemiological surveys assessing the effectiveness of elimination strategies, information derived from pooled sampling should also be considered for active case detection in low-transmission or pre-elimination areas to enable conducting large-scale surveys at a reasonable time and a reduced cost of molecular investigations in resource-limited countries. This approach has been proven to be cost-effective in surveillance studies, particularly when combined with real-time PCR [22–25]. However, molecular techniques that use high blood volumes are not suitable for large-scale surveillance studies due to logistic difficulties to collect and store venous blood under field conditions in tropical climates, compared to the more convenient collection storage and transport of small-volume dried blood spots on filter papers. Therefore, novel, field-deployable point-of-care approaches that combine blood pooling, samples of high blood volumes and LAMP should be developed and validated for the sensitive detection of submicroscopic malaria infections and for the characterization of drug resistance among parasite populations.

## Submicroscopic reservoir of resistant parasites: the missing link in malaria elimination strategies

Surveillance studies that examine antimalarial drug resistance are mostly concerned with the identification and validation of molecular markers of resistance in patients with laboratory-confirmed malaria [26], but disregard the detection of submicroscopic malaria infections. It is well documented that there are many more masked or hidden human infectious reservoirs in endemic areas than what can be detected by microscopy, and these reservoirs can contribute to maintaining malaria transmission and result in drug resistance emergence and/or spread [27–29]. Parasites resistant to antimalarial drugs and vectors resistant to insecticides are biological threats to malaria elimination [30–32]. This is why there are increasing concerns about the emergence and spread of artemisinin resistance in Southeast Asia, with the possibility of its spread to India and Africa [7], as this could possibly reduce the effectiveness of ACT interventions. Another concern is the dormancy, or temporarily arrested growth, of *P. falciparum* parasites that leads to some degree of tolerance to artemisinins [33–35]. This too may facilitate the emergence of parasite resistance to ACTs [34–36].

It is estimated that submicroscopic carriers are a source of 20–50% of all human-to-mosquito transmissions at very low levels of transmission [37], where submicroscopic gametocyte densities as low as <5 gametocytes/μl of blood can induce mosquito infections [27]. Early studies have shown that humans with microscopically undetectable gametocytes are infectious to mosquitoes, and that gametocyte and non-gametocyte carriers contribute to the infectious reservoir for malaria [38, 39]. Molecular techniques reveal that submicroscopic gametocytaemia is common after treatment [40], and such submicroscopic levels have been documented to contribute to mosquito infections after treatment with ACTs [41]. The question remains as to what extent submicroscopic infections contribute to the emergence and spread of resistance to antimalarial drugs, particularly in areas of low transmission. In such a context, it has been suggested that submicroscopic gametocytes following antimalarial treatment could be a possible marker of resistant parasites [42]. The magnitude and role of submicroscopic infections that are drug-resistant remain unknown. In surveillance studies, detection of submicroscopic reservoir of *P. falciparum* parasites is usually not accompanied by the estimation of the proportion of resistant parasite genotypes within this parasite population which may contribute to both malaria transmission and spread of antimalarial drug resistance. Together with other measures, it is of utmost importance to assess the hidden reservoir of resistant parasites and subsequently tailor suitable interventions for targeting it. Drug-resistant parasites persisting in a zone with continuous drug pressure have biological advantages that enhance infection and transmission, including better survival, increased proliferation of asexual erythrocyte stages and increased gametocyte transmissibility, compared to drug-sensitive parasites [43].

Several surveillance studies have used strategies such as detecting molecular markers of resistance among parasite isolates from malaria patients found positive by microscopy or from nucleic acids extracted from rapid diagnostic tests to study antimalarial drug resistance [44–46]. However, these approaches neglect the contribution of submicroscopic malaria infections to the reservoir of resistant parasites. Mathematical modeling predicts that mass screen-and-treat strategies can be successful in increasing the number of regions that can interrupt malaria transmission from an entomological inoculation rate of <1 to 1–4, if the diagnostic sensitivity increases from 200 to 2 parasites/μl [21].

Given that about half of all *P. falciparum* infections are not detected by microscopy in endemic areas [47], the drug-resistant parasite population is substantially underestimated. Although some studies have detected molecular markers of resistance after molecular diagnosis of *P. falciparum* malaria [48], this process is not routinely performed as part of surveillance systems in pre-defined sentinel sites. In addition, it ignores the detection of molecular markers in submicroscopic infections. The relative contribution of both microscopic and submicroscopic malaria infections to the total parasite population that are drug-resistant in a given area should be determined by tracking molecular markers of resistance in both types of infections. Such an integrated approach will determine the ratio of markers in submicroscopic infections to those in microscopic infections, through which the relative contribution of the submicroscopic reservoir of resistance to the total drug-resistant parasite populations in an endemic setting could be identified. Of particular importance is measuring the contribution of artemisinin resistance, as alternative drugs that can undertake artemisinin’s role in combination therapies are yet to be developed. Although several antimalarial drug candidates are in the pipeline to replace artemisinin, most are still being developed in exploratory clinical trials [49, 50].

Mapping the prevalence of molecular markers of drug-resistant malaria, particularly artemisinin derivatives, in submicroscopic and microscopic infections in endemic areas is urgently needed. The recent discovery of a molecular marker of artemisinin resistance due to mutations in the kelch gene *K13* [51] could facilitate the mapping of resistance at its molecular level in submicroscopic and microscopic falciparum malaria before these infections become clinically evident. The containment of artemisinin resistance through enhanced and sustained surveillance of resistant parasites should be given top priority [52]. In addition, the novel epidemiological tool Focused Screening and Treatment has been recently introduced for active molecular detection, treatment and tracking of asymptomatic parasite carriers to counteract the spread of drug-resistant parasites [53]. This supports the need for the development and validation of new tools that can effectively detect and track submicroscopic foci of resistant parasites in areas approaching malaria elimination. Interventions for malaria elimination can thus be based on evidence-based knowledge of the relative contribution of submicroscopic malaria infections to the total reservoir of resistant parasites.

Molecular surveillance of resistance markers, which is mostly based on the detection of parasites in microscopic infections, should be extended to detection and characterization of submicroscopic infections in future strategies for malaria elimination. This will shift the surveillance of antimalarial drug resistance from reactive to proactive actions. Therefore, if resistance emerges in a defined geographical area, even at the submicroscopic level, appropriate interventions can be undertaken to eliminate the detected foci of resistance. For example, the gametocytocidal drug primaquine could be administered to the population in that area, according to the updated recommendation of the WHO’s Malaria Policy Advisory Committee, in order to block the transmission of both drug-sensitive and drug-resistant parasites to vectors [54].

## Conclusions

The integrated tracking of molecular markers of resistance in submicroscopic and microscopic malaria infections could enhance the detection of emerging drug resistance, as well as identify the “true” burden of resistant parasites in settings that have different malaria endemicities. Surveillance studies that examine the hidden reservoir of resistant parasites in low-transmission settings should enhance their sensitive detection methods; first, extracting the parasite’s nucleic acids from a large amount of blood (0.5–3 ml), followed by detection using ultra-sensitive real-time PCR [55]. Of equal importance is the detection and monitoring of reservoirs of submicroscopic resistant parasites in sentinel sites in malaria-endemic areas. In addition, the contribution of these reservoirs to the spread of malaria transmission and drug resistance in such settings should be determined in the context of malaria elimination efforts. Steps should also be taken to develop new highly-sensitive, field-applicable molecular approaches for detecting resistant parasite genotypes responsible for submicroscopic infections.

## Additional file

Additional file 1: Multilingual abstracts in the five official working languages of the United Nations. (PDF 791 kb)

## Abbreviations

ACT: Artemisinin-based combination therapy
ITN: Insecticide-treated net
LAMP: Loop-mediated isothermal amplification
PCR: Polymerase chain reaction
UN: United Nations
WHO: World Health Organization
