# Supplementary material for: Hidden reservoir of resistant parasites: the missing link in the elimination of falciparum malaria
Source: Infect Dis Poverty. 2017 Feb 6;6:12. doi: 10.1186/s40249-016-0227-5 (PMC5294824; doi:10.1186/s40249-016-0227-5)
Supplement: Additional file 1: — Multilingual abstracts in the five official working languages of the United Nations. (PDF 791 kb) [file 40249_2016_227_MOESM1_ESM.pdf]

Translation of the abstract into the five official working languages of the United Nations

مصدر غير ملاحظ للطفيليات المقاومة: الحلقة المفقودة في القضاء على الملاريا المنجلية

رشاد عبد الغني، محمد أ. ك. مهدي، جون س. بيير، ليوناردو ك. باسكو

ملخص

خلفية: للقضاء على الملاريا يجب إحداث نظام متكامل يتضمن عددا من أساليب التعامل والتدخلات الرامية إلى التغلب على تهديد مقاومة الأدوية المضادة للملاريا. وقد تم إحراز تقدم كبير في الحد من معدلات الإصابة بمرض الملاريا من خلال استخدام نطاق واسع من العلاجات التوليفية القائمة على مادة الأرتيميسينين والناموسيات المعالجة بمبيدات الحشرات. ولتعزيز هذه المكاسب، ينبغي إيلاء الانتباه إلى الحلقات المفقودة في القضاء على الملاريا. واحدة من هذه الثغرات هي الخزان المتبقي من الطفيليات المقاومة دون المجهرية، التي بقيت بعد أن تم تنفيذ إجراءات المكافحة أو اتخاذ تدابير المكافحة الأخرى بها. ولذلك، فإن هذه الدراسة تسلط الضوء على أهمية استكشاف الدور الذي يمكن أن تلعبه الطفيليات المقاومة دون المجهرية في إعاقة القضاء على الملاريا من خلال السماح باستمرار انتقالها، ولا سيما في الأماكن التي تكون فيها معدلات الانتقال منخفضة أو في مرحلة ما قبل القضاء و / أو مرحلة القضاء على مصدر العدوى.

المناقشة: حتى تكون أساليب التعامل المتبعة للتخلص من الملاريا فعالة، والدور النسبي للمصدر غير الملاحظ للطفيليات المقاومة يحتاج إلى تقييمه، لا سيما في المناطق التي تكون فيها معدلات الانتقال منخفضة و / أو في مرحلة ما قبل القضاء و / أو مراحل القضاء على مصدر العدوى. وتركز الدراسات الجارية المختلفة حول دور الإصابات بمرض الملاريا دون المجهرية في انتقال الملاريا ولكن نتغاضى عن احتمال تراكم مقاومة للعقاقير المضادة للملاريا بين مجموعات الطفيل تحت المجهري. وهذا عامل مهم لأنه قد يحد في نهاية المطاف من فعالية استراتيجيات القضاء على الملاريا.

الاستنتاجات: التقدير المبني على الأدلة للمصدر "الحقيقي" للطفيليات المقاومة يمكن أن يساعد في استهداف البؤر القائمة والناشئة من الطفيليات المقاومة قبل انتشارها. وظهور وانتشار ملاريا المتصورة المنجلية المقاومة للأرتيميسينين في جنوب شرق آسيا يؤكد الحاجة لاحتواء المقاومة للأدوية.

Translated from English version into Arabic by Mahmoud Sami, through

TRANSLATORS
WITHOUT BORDERS

抗药性疟原虫的隐蔽宿主：消除恶性疟的缺失环节

Rashad Abdul-Ghani, Mohammed A. K. Mahdy, John C. Beier, Leonardo K. Basco

摘要

引言：成功消除疟疾需要一个综合的体系，包括以克服抗疟药抗性威胁为目标的一系列方法和干预措施。在降低疟疾发病率方面，通过大规模使用以青蒿素为基础的联合疗法和杀虫剂浸泡蚊帐，已取得了显著成效。为了巩固这些成果，需要更加注意在消除疟疾过程中的某些缺失环节。缺口之一就是低密度的抗性疟原虫的储存宿主，虽然经过病例管理或其他控制措施，但是低密度原虫仍然存在。因此，本研究强调探索低密度抗性疟原虫在阻碍消除疟疾中的作用的重要性，因为它可使疟疾持续传播，特别是在一些低水平传播，或在消除前和处于消除阶段的地区。

讨论：如果疟疾消除干预措施是有效的，那么就应该评估隐蔽保虫宿主的相对作用，特别是一些在低水平传播，或在消除前和处于消除阶段的地区。有很多研究将重点放在研究低密度疟疾感染在疟疾传播中的作用，但是却忽略了低密度疟原虫群体抗药性的逐渐增加，而这可能是一个最终影响疟疾消除策略的重要因素。

结论：以证据为基础估算“真正”的抗性疟原虫储存宿主可以帮助在抗性扩散之前定位现有的和新出现的抗性位点。东南亚恶性疟原虫对青蒿素抗药性的出现和传播进一步强调了需要控制抗疟药物的抗性。

Translated from English version into Chinese by Xin-Yu Feng, edited by Pin Yang, through
CHINESE CENTER FOR DISEASE CONTROL AND PREVENTION
NATIONAL INSTITUTE OF PARASITIC DISEASES

Réservoir caché des parasites résistants: le chaînon manquant dans l'éradication du paludisme à *falciparum*

Rashad Abdul-Ghani, Mohammed A. K. Mahdy, John C. Beier, Leonardo K. Basco

Résumé

Historique: Pour éradiquer le paludisme avec succès, un système intégré comportant un certain nombre d'approches et d'actions, visant à faire reculer la résistance aux médicaments antipaludiques – est nécessaire. Des avancées significatives ont été effectuées pour réduire l'incidence du paludisme à travers une utilisation à grande échelle de polythérapies à base d'artémisinine et de moustiquaires imprégnées d'insecticide. Pour consolider ces acquis, il convient de porter une attention particulière aux chaînons manquants dans l'éradication du paludisme. Une de ces lacunes est le réservoir résiduel des parasites sous-microscopiques résistants qui demeure, après que la gestion des cas ou autres mesures de contrôle aient été effectuées. Le présent article souligne ainsi l'importance d'examiner le rôle que pourraient jouer les parasites sous-microscopiques résistants à entraver l'éradication du paludisme en favorisant la persistance de la transmission, particulièrement dans les régions où le taux de transmission est faible ou lors des phases de pré-élimination ou d'élimination.

Débat : Pour que les actions en matière d'éradication du paludisme soient efficaces, il est nécessaire d'évaluer le rôle relatif du réservoir caché des parasites résistants, en particulier dans les régions où les taux de transmission sont faibles et/ou lors des phases de pré-élimination ou d'élimination. De nombreuses études en cours sont axées sur le rôle des infections sous-microscopiques du paludisme en terme de transmission de la maladie mais elles négligent l'accumulation possible de résistance aux médicaments antipaludiques au sein des populations des parasites sous-microscopiques. Ceci est un facteur important puisqu'il peut éventuellement restreindre l'efficacité des stratégies en matière d'éradication du paludisme.

Conclusions : Une estimation concrète du "réel" réservoir de parasites résistants peut aider à cibler le foyer existant et émergent des parasistes résistants avant qu'ils ne se propagent. L'émergence et la propagation de la souche *Plasmodium falciparum* résistante à l'artémisinine en Asie du Sud-Est rappelle la nécessité de maitriser la résistance aux médicaments.

Translated from English version into French by veromarie, through
TRANSLATORS
WITHOUT BORDERS

Скрытый источник устойчивых паразитов: недостающее звено в ликвидации малярии falciparum

Рашад Абдул-Гани, Мохаммед А. К. Махди, Джон К. Байер, Леонардо К. Баско

Реферат
Предпосылки: Для успешной ликвидации малярии требуется интегральная система, включающая несколько подходов и вмешательств, направленных на преодоление угрозы резистентности к антималярийным препаратам. Значительный прогресс в снижении встречаемости малярии был сделан благодаря широкомасштабному использованию комбинированных терапий на основе артемизинина и обработанных инсектицидами сеток. Чтобы консолидировать этих достижения, необходимо уделить внимание недостающим звеньям в ликвидации малярии. Одной из лакун является остаточный источник из субмикроскопических резистентных паразитов, остающийся после обработки конкретного случая и других мер контроля. Поэтому в настоящем выраженном мнении освещена важность изучения той роли субмикроскопических резистентных паразитов, которую они могут играть в препятствовании ликвидации малярии, способствуя сохранению распространения, в особенности в районах в низким распространением или на фазе предликвидации и/или ликвидации.
Обсуждение: Чтобы вмешательства по ликвидации малярии были эффективными, необходимо оценить относительную роль скрытого источника резистентных паразитов, особенно в районах, являющихся районами с низким распространением и/или в предликвидационной и/или ликвидационной фазах. В различных ведущихся исследованиях рассматривается роль субмикроскопических малярийных инфекций в передачи малярии, но упущено возможное нарастание резистентности к противомалярийным препаратам в субмикроскопических популяциях паразитов. Это важный фактор, могущий в конечном итоге ограничить эффективность стратегий по борьбе с малярией.
Выводы: Оценка на основании свидетельств «истинного» источника резистентных паразитов может помочь нацелиться на имеющиеся и формирующиеся средоточия резистентных паразитов до их распространения. Выход и распространение устойчивой к артемизинину малярии *Plasmodium falciparum* в Юго-Восточной Азии подчеркивает необходимость сдерживания лекарственной резистентности.

Translated from English version into Russian by Alexander Somin, through

TRANSLATORS
WITHOUT BORDERS

Reservorio oculto de parásitos resistentes: el eslabón perdido en la eliminación del paludismo por P. Falciparum

Rashad Abdul-Ghani, Mohammed A. K. Mahdy, John C. Beier, Leonardo K. Basco

Resumen
Antecedentes: Para eliminar el paludismo con éxito se requiere un sistema integrado que

incluya diferentes enfoques e intervenciones encaminados a combatir la amenaza que supone la resistencia a los medicamentos antipalúdicos. Se ha avanzado significativamente en la reducción de la incidencia del paludismo gracias a la utilización a gran escala de tratamientos combinados a base de artemisinina y al uso de mosquiteros tratados con insecticida. Para consolidar estos progresos se debe prestar atención a los eslabones perdidos en la eliminación del paludismo. Una de estas lagunas es el reservorio residual de parásitos submicroscópicos resistentes que perviven tras haber realizado la gestión de un caso o haber aplicado otras medidas de control. El presente articulo de opinión subraya, en consecuencia, la importancia de la investigación del papel de los parásitos submicroscópicos resistentes como obstáculo de la eliminación del paludismo al permitir la persistencia de la transmisión, particularmente en áreas de baja transmisión o en las fases de preeliminación y/o eliminación.

Discusión: Para que las intervenciones de eliminación del paludismo sean efectivas, se debe evaluar el papel relativo del reservorio oculto de parásitos resistentes, especialmente en zonas de baja transmisión y/o en las fases de preeliminación y/o eliminación. Varios estudios en curso se centran en el papel de las infecciones de paludismo submicroscópicas en la transmisión del paludismo pero pasan por alto el posible desarrollo de resistencia a los medicamentos antipalúdicos entre las poblaciones de parásitos submicroscópicos. Este es un factor importante ya que puede eventualmente limitar la efectividad de las estrategias de eliminación del paludismo.

Conclusiones: Una valoración con base empírica del "verdadero" reservorio de parásitos resistentes puede ayudar a atacar los focos existentes y emergentes antes de que se propaguen. La aparición y propagación de *Plasmodium falciparum* resistente a la artemisinina en el sudeste asiático recalca la necesidad de evitar la difusión de la farmacorresistencia.

Translated from English version into Spanish by Sergio Campo, through

TRANSLATORS
WITHOUT BORDERS
